# Unravelling the molecular landscape of polycystic ovary syndrome (PCOS) and role of inflammation through transcriptomics analysis of human ovarian granulosa cells

**DOI:** 10.1186/s44342-025-00051-6

**Published:** 2025-08-11

**Authors:** Kanika Mahra, Vineet Singh, Jae-Ho Shin

**Affiliations:** Department of Applied Biosciences, Kyungpook National University, Daegu, 41566 Republic of Korea

**Keywords:** RNA-seq, Granulosa cells, Inflammation, PCOS, Obesity

## Abstract

**Background:**

Polycystic ovary syndrome (PCOS) is a common metabolic problem in women of reproductive age that can lead to infertility and other metabolic disorders. Recent evidence indicates that inflammation might be one of the contributing factors in PCOS progression. However, there is a lack of information on the regulation of inflammatory genes in PCOS. Therefore, the aim of the study is to investigate the role of inflammation-associated genes and pathways in relation to PCOS.

**Method:**

The bulk RNA-seq data of granulosa cells of human ovaries of PCOS-affected and healthy women were analyzed to evaluate the inflammatory regulation in PCOS. After quality trimming, the raw RNA-seq data were aligned to the human genome, and gene expression was quantified using featureCounts with Ensembl annotation. Further, downstream analyses of the resulting count matrix were performed in R Studio, where differentially expressed genes (DEG) were identified and CO-DEG analysis was performed.

**Results:**

The study identifies the various differentially expressed inflammatory genes in the case of PCOS such as SPI1, HSPB1, MNDA, and ITGA. These DEG are closely associated with the activation of inflammatory responses, i.e., activation of lymphocytes and leukocytes, leukocyte migration and mononuclear cell proliferation, stimulating binding of various cytokines, immunoglobulins, and chemokines. PCOS group also exhibited an increased expression of androgen-mediated genes (SPI1 and ETS transcription factors) and genes associated with hyperlipidemia and insulin resistance (TNFRSF1B). Further, KEGG pathway enrichment analysis revealed significant upregulation of various pathways (autophagy, endocytosis) in the PCOS group. In addition, network analysis (cnetplot) of the top 10 KEGG GSEA pathways also highlights the key pathways in the PCOS group such as SNARE complex assembly pathway, SNAP-25, nucleophagy, and regulation of mast cell activation.

**Conclusion:**

Therefore, the study highlights that inflammation is a major effector in PCOS, which also fuels obesity, an independent effector that further worsens the PCOS condition. In addition, the genes related to hyperandrogenism, hyperlipidemia, and insulin resistance were also overexpressed in PCOS, exacerbating the condition.

**Supplementary Information:**

The online version contains supplementary material available at 10.1186/s44342-025-00051-6.

## Introduction

Polycystic ovary syndrome (PCOS) is a complex, multifactorial metabolic-endocrine disorder that significantly affects the health of women between the age of 16–40 years. According to the World Health Organization (WHO), PCOS affects 6–13% of women of reproductive age globally, with approximately 70% of cases remaining undiagnosed. The primary symptoms of PCOS include hormonal imbalance, irregular menstrual cycles, ovarian cysts, hyperandrogenism, and anovulation [1]. The etiology of PCOS is multifaceted and includes genetic predisposition (family history), environmental factors (such as an inactive lifestyle and diet), and obesity. PCOS is associated with a wider range of several metabolic, including type 2 diabetes, cardiovascular disease, non-alcoholic fatty liver disease (NAFLD), hypertension, dyslipidemia, and various pregnancy-related complications [2]. Women with PCOS have a higher prevalence of pregnancy-induced hypertension, gestational diabetes, and pre-eclampsia [3]. A large meta-study revealed that women with PCOS have higher obstetric complications, while non-obese women with PCOS have a higher risk of developing gestational hypertension, and gestational diabetes [4]. In addition, women with PCOS have a higher chance to develop endometriosis by altering the functioning of the hypothalamic-pituitary–gonadal (HPG) axis [5], and endometrial cancer as compared to non-PCOS [6].

The pathogenesis of PCOS is associated with chronic and low-grade inflammation, which promotes and complicates the outcome of PCOS [7]. Individuals with PCOS consistently exhibit chronic low-grade inflammation marked by increased levels of pro-inflammatory cytokines, including C-reactive protein (CRP), interleukin-6 (IL-6), and tumor necrosis factor-α (TNF-α) [8]. Studies have shown that dysregulation of the pathways can result in higher levels of NF-κB and increased levels of reactive oxygen species (ROS) [9]. A mouse study also suggested the differential expression of genes related to inflammatory responses, showing upregulation of genes related to inflammation such as Cd19, Nlrc4, Nox4, and Gzmb, and inflammation-related gene products [10]. However, there is a lack of direct information on the molecular mechanisms and inflammatory pathways involved in PCOS among women of reproductive age. This can be assessed through gene expression analysis of granulosa cells, which play a key role in follicular development and hormone regulation and are significantly affected in PCOS, making them a critical target for investigation. In addition, transcriptomic profiling allows for the identification of differentially expressed genes (DEG) and dysregulated pathways, providing a clearer understanding of the genetic factors influencing metabolic dysfunction, inflammation, and hormonal imbalance in PCOS. Given the complex interplay between hormonal imbalance, inflammation, and metabolic dysfunction in PCOS, transcriptomic analysis is essential to uncover the gene expression changes that drive the disorder. Transcriptomics (RNA-Seq) can provide deeper insights into the molecular mechanisms underlying PCOS.

Therefore, in the present study, we analyzed bulk RNA-seq data of human ovarian granulosa cells. Granulosa cells, which play a key role in follicular development and hormone regulation, are significantly affected in PCOS, making them a critical target for investigation. Transcriptomic analysis was used to identify the DEG and dysregulated pathways, providing a clearer understanding of the genetic factors influencing metabolic dysfunction, inflammation, and hormonal imbalance in PCOS. This approach enhances our understanding of the pathophysiology of PCOS and also helps to identify potential therapeutic targets to mitigate its clinical impact.

## Material and methods

### Selection criteria and data retrieval

We conducted a comprehensive search of the National Center for Biotechnology Information (NCBI) database using Medical Subject Headings (MeSH) terms, both in combination and independently, to identify relevant studies. Specific search terms included “polycystic ovarian syndrome”, “PCOS”, “polycystic ovarian morphology”, “granulosa cell”, “granulosa cell and PCOS”, “PCOS transcriptomics”, and PCOS RNAseq”. Using these keywords, we screened and selected target studies for further analysis. For RNAseq data, we focused exclusively on human PCOS and ovarian granulosa cell samples, so no mouse or animal samples were included in the analysis. The samples included in the study were from premenopausal women diagnosed with PCOS according to the Rotterdam criteria, which require at least two of the following: oligo/anovulation, clinical/biochemical hyperandrogenism, and polycystic ovarian morphology [11]. Exclusion criteria included any participant with hyperprolactinemia; thyroid dysfunction; abnormal liver, kidney, or heart function; gastrointestinal disease; diabetes; recent use of oral contraceptives, steroids, or antibiotics; or who had been treated for PCOS, pregnant, or lactating within the past year. The required RNA sequences (Sequence Read Archive, SRA; accession numbers, SRR) were selected from the identified research papers. A total of 19 samples meeting the above inclusion criteria were included in the study, and detailed sample information is provided in Table S6. The raw sequencing data were then retrieved from the NCBI SRA using the SRA Toolkit for further analysis.

### Data processing and analysis

**Fig. 1:**
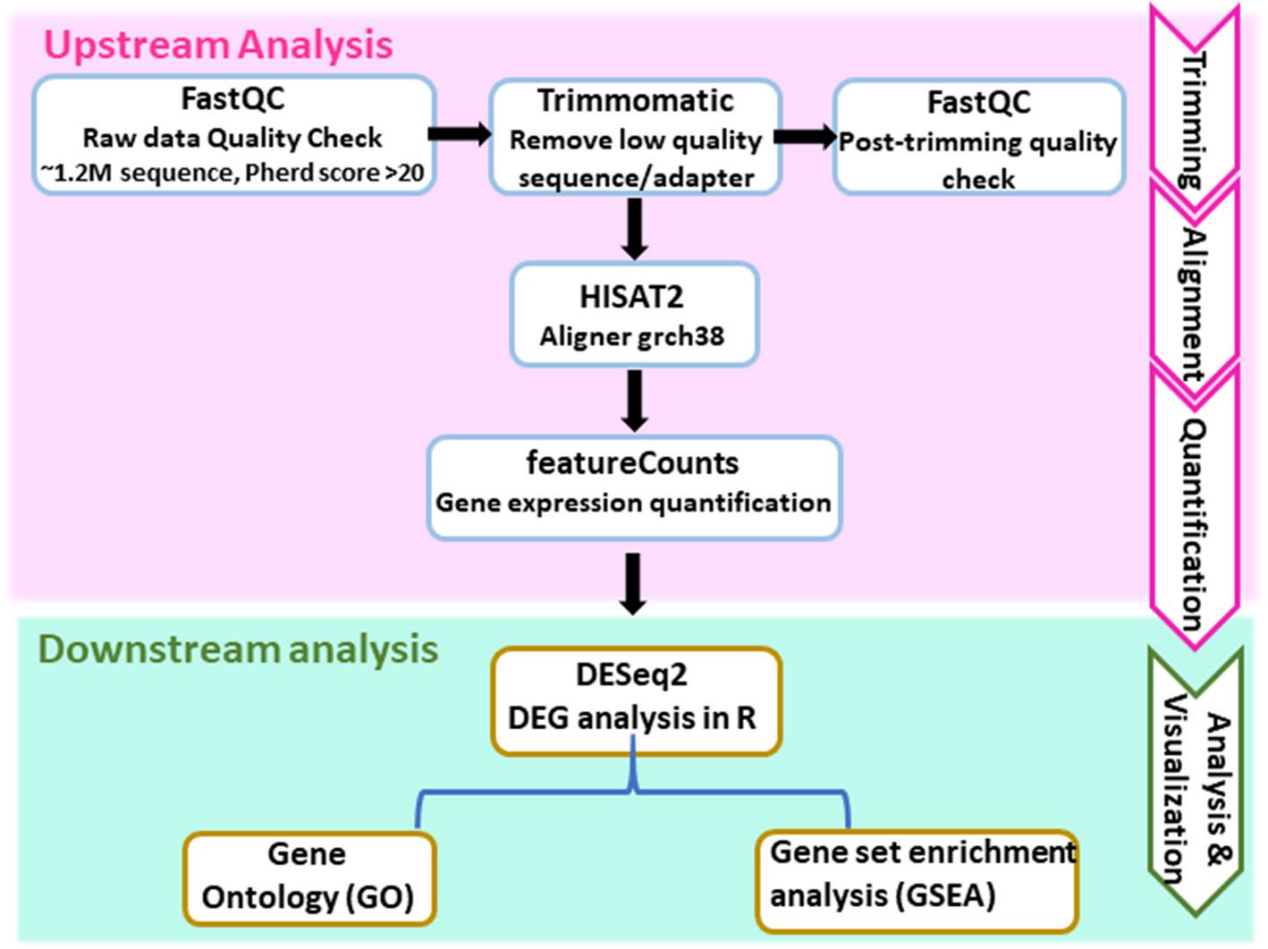
Work-flow for the analysis

The quality of the raw RNA sequence data was assessed using FastQC (v0.11.9). On average, each sample contained approximately 1,250,000 reads, with no quality flags raised. The per-base Phred quality scores remained consistently above 20 across most of the reads, indicating high sequencing quality. The resulting reads were trimmed using Trimmomatic v0.39 to remove low-quality bases, with parameters for end trimming (leading 3, trailing 3), sliding window trimming (slidingwindow 4:15), and minimum read length filtering (minimum length 25). After trimming, the total reads were 42,140,211,6 (ranging from 9,766,246 to 37,697,435). The total number of reads obtained post-trimming was 37,634,739,7 (89.31%), ranging from 99.92 to 78.29%. The quality of the trimmed reads was further ensured by FastQC. These high quality reads were then aligned to the human genome assembly (grch38) using HISAT2 (v2.2.0) [12]. The resulting SAM files were processed with Samtools and converted to BAM files [13]. Then, gene expression was quantified as a count matrix using featureCounts, which assigned mapped reads to features based on the Ensembl genome annotation [14]. Further, downstream analyses of the count matrix were performed in R studio (Fig. 1).

### Analysis of differentially expressed genes and enriched pathways

For RNA-seq analysis, feature counts were used for further statistical analysis in R (version 4.3.2). Differential expression (DE) analysis was conducted using the DESeq2 R package (version 1.42.1) [15]. The DESeq function was used for differential gene expression analysis, with the Wald test applied as the default statistical method. DEGs were identified using the following criteria: adjusted *p*-value < 0.05, baseMean > 100, and |log2 fold change|≥ 1.5, *p*.adj < 0.05. Gene expression data were normalized using *z*-scores (mean = 0, standard deviation = 1) to facilitate better visualization of differential expression. The results were visualized using a volcano plot, of the top 30 most significant (*p* < 0.05) genes. Principal component analysis (PCA) was also generated to visualize the gene expression variation. Later, Co-DEGs analysis including gene ontology (GO) enrichment and pathway enrichment, is performed using the enrichGO function of the ClusterProfiler (v.4.10.1) package in R, taking only genes with selected criteria (baseMean > 100, log2FoldChange > 1.5, padj < 0.05). The GO enrichment analysis was performed on the top 30 biological processes (BP), molecular funtion (MF), and cellular component (CC) based on the adjusted *p*-value using the clusterProfiler package. Overrepresentation analysis of DEG was also performed using enrichKEGG to identify enriched pathways in the predefined DEG list. The gene list for this analysis was derived from DEGs identified in the RNA-seq dataset. Further, KEGG pathway enrichment analysis was conducted using the gseKEGG function from the clusterProfiler R package (v.4.10.1) with the following parameters: nPerm = 100,000, minGSSize = 3, maxGSSize = 800, pvalueCutoff = 0.05, pAdjustMethod = “none”, and keyType = “ncbi-geneid’. The gene set enrichment analysis (GSEA) was conducted using gseGO function and, cnetplot was used to visualize the relationships between the top 10 enriched KEGG pathways and associated genes, highlighting the functional relationship and common genes among pathways.

## Results

### Quality control and data overview

**Fig. 2:**
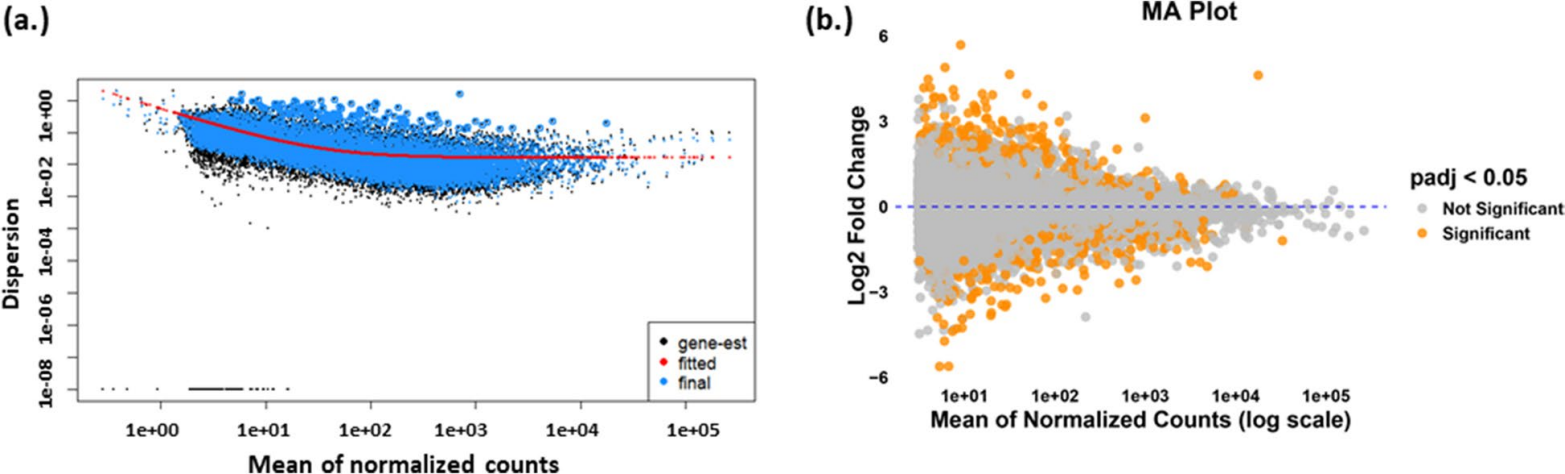
**a** Dispersion plot showing the dispersion trend which decreases smoothly with increasing gene expression, leveling off at a plateau that reflects the biological variability in the dataset for a typical gene. **b** The MA (log ratio vs. mean) plot showing genes that are differentially expressed displays the moderated log-fold change on the *y*-axis and the mean of normalized counts on the *x*-axis. Orange points represent genes identified as DEG, based on a nominal false discovery rate threshold of 0.05

The quality check by FastQC showed that prior to processing, approximately 1,250,000 sequences with no poor-quality flags and consistently high per-base Phred scores above 30 were used for analysis. In the pre-processing step, after filtering the DESeq2 dataset, the number of features was reduced from 63,144 to 19,315, with a total read count of 233, 289, 429, and no singletons were detected (Table S1 and S2). The DESeq2 result summary indicated that 3.4% of genes were upregulated, 4.4% were downregulated, and 5.8% of genes had low counts, with no outliers detected. The dispersion plot of the normalized read count (Fig. 2a) and the mean vs. average (MA) plot showed the dispersion and DEG, respectively (Fig. 2b). Initially 847 DEGs were detected, but after filtering the number was reduced to 45 (Table S3 and S4).

### Gene cluster and identification of differentially expressed genes

**Fig. 3:**
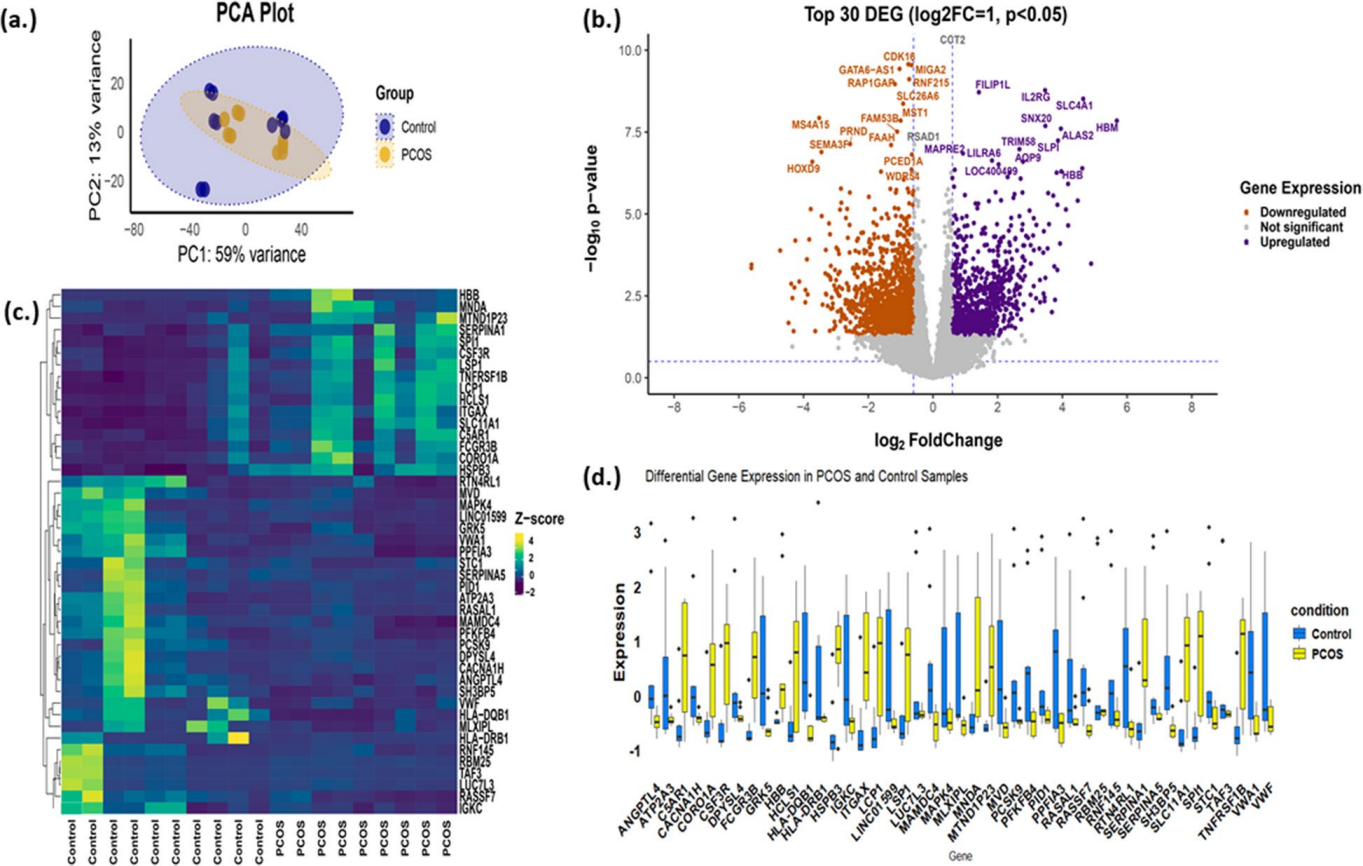
**a** PCA plot of PCOS vs control. **b** Volcano plot of up-and down-regulated DEG in PCOS, showing top 30 DEG.** c** Heatmap of top 45 DEG. **d** Box plot of same 45 DEG simultaneous comparison

The PCA analysis showed the different gene expression between the control and PCOS groups (Fig. 3a). In addition, the top 30 DEGs were also highlighted, including both upregulated (HBM, ALAS2, SLC4A1, HBB) and downregulated (SEMA3F, MS4A15, GATA-AS1, FAAH, PRND, etc.) genes in the PCOS group compared to the control (Fig. 3b). Analysis of the selected DEGs in both the PCOS and control groups showed that the top 45 DEGs revealed that the expression of genes related to inflammation was upregulated in the PCOS group (Fig. 3c, d). In the PCOS group, eight inflammatory genes (SPI1, TNFRSF1B, CSF3R, SERPINA1, HSPB1, SLC11A1, ITGAX, MNDA) were differentially expressed.

### Gene ontology (GO) and pathway enrichment analysis of PCOS

**Fig. 4:**
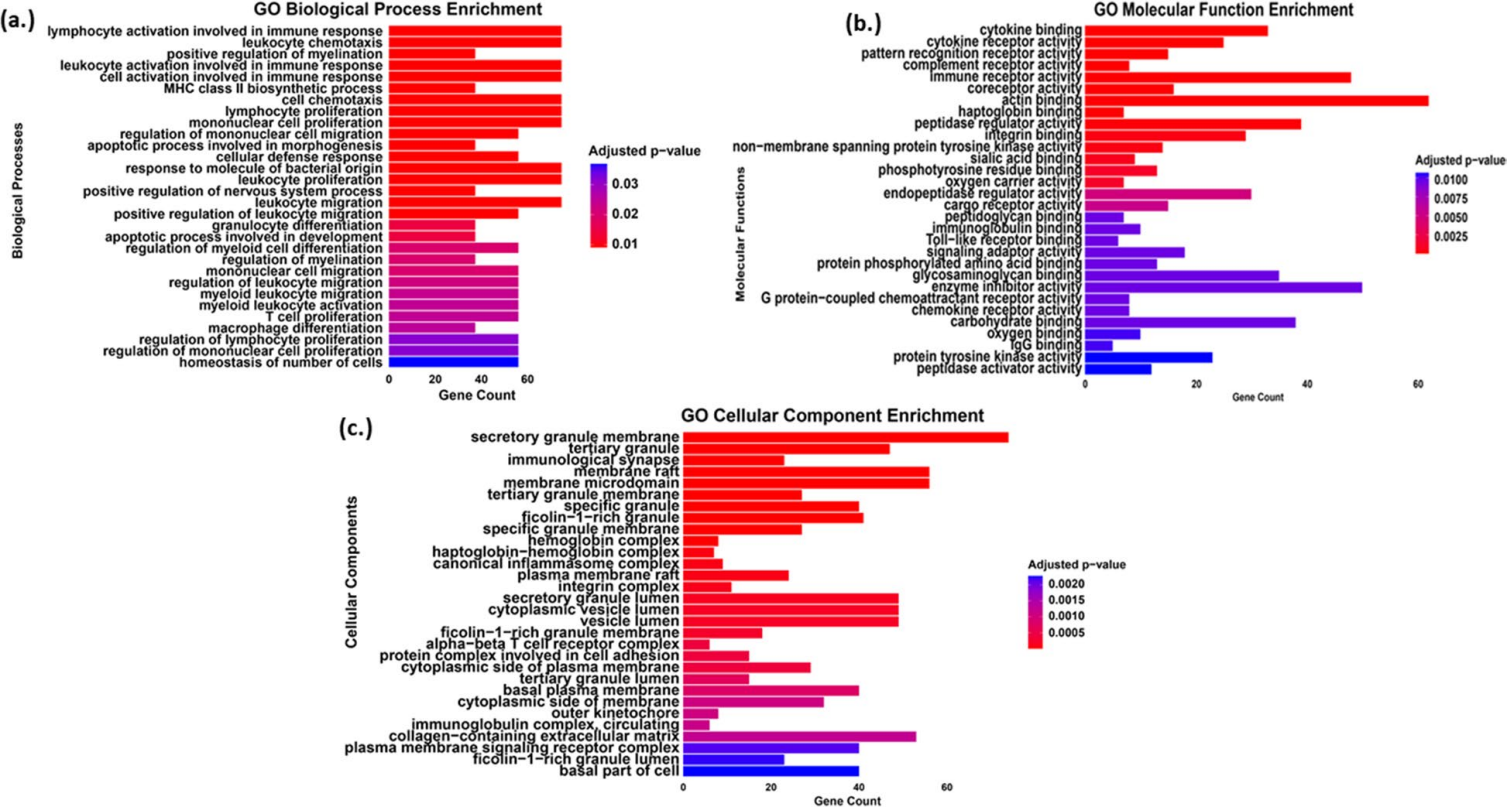
**a** Top 30 biological processes (BP) of gene ontology in PCOS group. **b** Top 30 molecular functions (MF) of gene ontology in PCOS group. **c** Top 30 cellular components (CC) of gene ontology in PCOS group

The GO analysis was performed using enrichGO on significantly expressed genes (Table S5). The enrichGO was then used to analyze the biological process (BP), molecular function (MF), and cellular component (CC) categories of the DEGs. Amongst the top 30 highly significant of all terms (BP, MF, and CC) were used to visualize in horizontal bar plots (Fig. 4a–c). The GO-BP analysis highlights the BP that was predominantly associated with immune system imbalance and chronic inflammation. The key pathways identified include lymphocyte and leukocyte activation in immune responses, lymphocyte and leukocyte proliferation, and cell and leukocyte chemotaxis. Similarly, GO-MF enrichment analysis reveals molecular functions primarily associated with immune activation and inflammation, highlighting a strong inflammatory component (Fig. 4b). The significant involvement of cytokine signaling, toll-like receptor pathways, and chemokine activity suggests an elevated immune response, potentially associated with chronic inflammation. In addition, GO-CC enrichment analysis revealed key inflammation-associated components, including the immunological synapse, canonical inflammasome complex, plasma membrane signaling receptor complex, and the secretory granule membrane, indicating an active inflammatory response (Fig. 4C).

### KEGG pathway enrichment analysis in PCOS

**Fig. 5:**
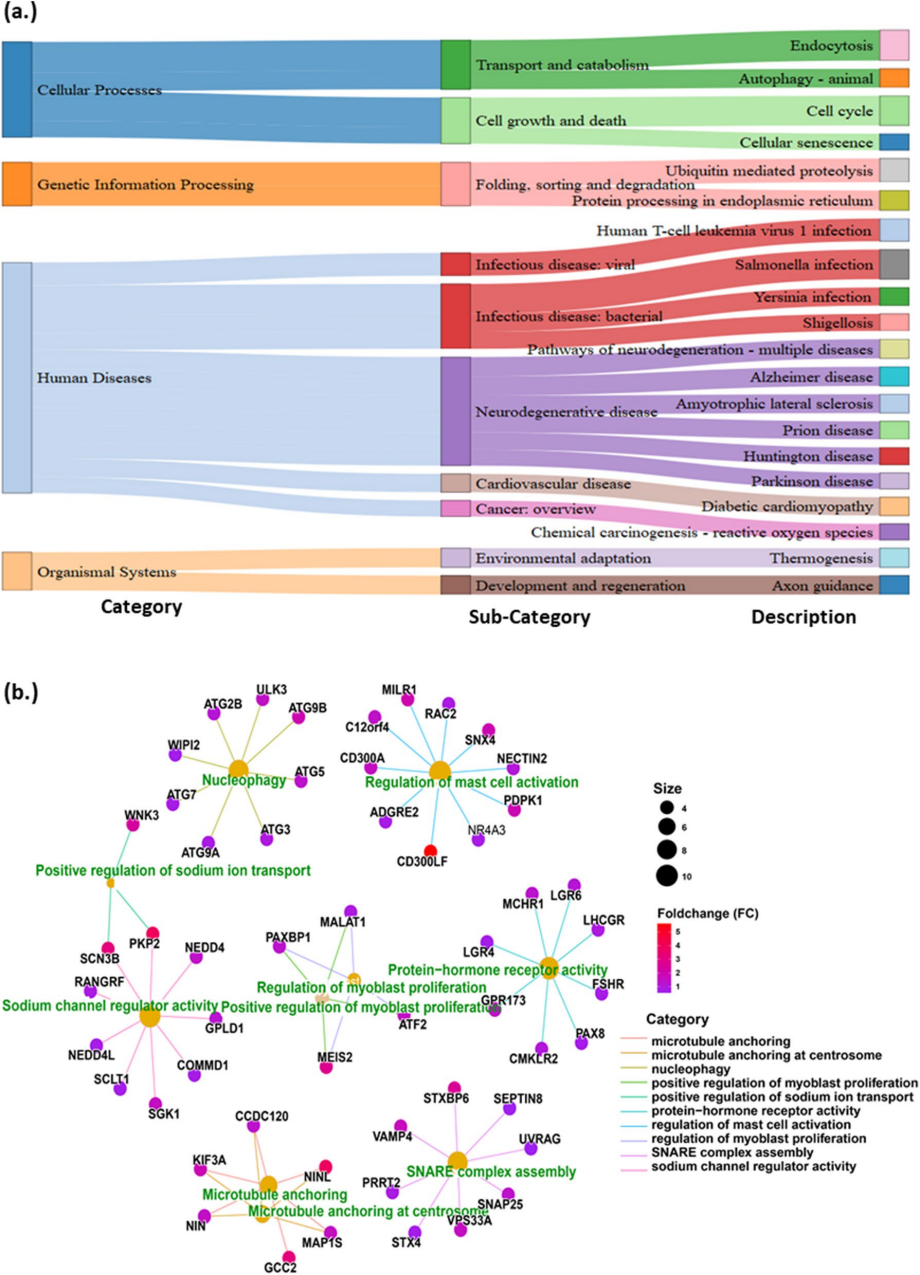
**a** Top 20 KEGG pathways in PCOS group in DEG.** b** Circular network plot (cnetplot) of top 10 significantly enriched KEGG pathways and genes associated with those pathways

KEGG pathway enrichment analysis revealed significant upregulation of various pathways in the PCOS group, including chemical carcinogenesis–reactive oxygen species, autophagy, endocytosis, and ubiquitin-mediated proteolysis. The PCOS group also highlighted the pathways related to human diseases such as infectious disease, cardiovascular disease, and neurodegenerative disease pathways, indicating potential systemic inflammation in PCOS (Fig. 5a). Further, the network analysis (cnetplot) of the top 10 KEGG GSEA pathways also highlights the key gene-pathway interactions (Fig. 5b). Notably, the SNARE complex assembly pathway was enriched with essential genes such as SEPTIN8, SNAP25, and STX playing a crucial role. Similarly, the protein hormone receptor activity pathway is associated with key genes including FSHR, LHCGR, LGR6, and LGR4, which are critical for hormone signaling and regulation. Another important pathway includes the regulation of mast cell activation, nucleophagy, and positive regulation of myoblast proliferation.

## Discussion

### Quality control and analysis of differentially expressed genes (DEG)

The dispersion plot shows a decreasing trend in dispersion with increasing gene expression, stabilizing at a plateau reflecting biological variability, which indicates higher variability in low-expressed genes. Additionally, the MA plot identifies DEG (FDR < 0.05), with significant log-fold changes mainly observed at lower expression levels. Overall, this suggests that low-expressed genes are more variable and more likely to show significant differential expression, while highly expressed genes are more stable in their expression levels. The PCA plot of gene expression between the normal and PCOS groups was also a clear indication of the differences.

Further, in the PCOS group, DEG analysis revealed downregulation of the SEMA3F gene, which may impair GnRH neuron migration, disrupt reproductive hormone signaling, and contribute to the hormonal imbalances associated with the disorder [16]. The lower expression of fatty acid amide hydrolase (FAAH) in women with PCOS has also been noted previously [17]. Similarly, there is a downregulation of the GATA6-AS1 gene, and its reduced expression was also reported in IBD, Crohn’s disease, celiac disease [18]. In addition, ovarian granulosa cells under PCOS conditions were also found to have upregulated Alas2, which contributes to oxidative stress, apoptosis, and mitochondrial dysfunction [19]. Further, DEGs that were upregulated in PCOS groups like SPI1 and ETS-transcription factor regulate androgen-mediated gene expression in PCOS [20], potentially contributing to the increased androgen production. Similarly, upregulated TNFRSF1B was reportedly strongly associated with hyperandrogenism in PCOS and metabolic disorders, including insulin resistance, hypertension, and hyperlipidemia, highlighting the role of inflammatory cytokines in the pathogenesis of PCOS [21]. In addition, various genes related to inflammation were also upregulated in the PCOS group, such as ITGAX, a pro-inflammatory macrophage gene encoding an integrin αX chain, which is linked to hypertriglyceridemia, hypercholesterolemia, and atherosclerosis [22, 23]. Similarly, elevated CSF3R expression in the PCOS group indicates higher inflammation and promotes granulopoiesis, thus contributing to the chronic inflammatory state associated with the disorder [24]. Additionally, in the PCOS group, increased expression of SERPINA1 and SLC11A1 was found to be associated with insulin resistance and inflammation, respectively [22, 25]. Thus, the analysis highlights that genes related to inflammation and metabolic disorders were overexpressed in the PCOS condition, which was consistent with the previous findings [2].

### Gene ontology of PCOS

The GO enrichment analysis of significantly expressed genes in the PCOS group further revealed a strong association with inflammation and immune dysregulation. As the GO analysis of the biological process (BP) group, genes were mainly enriched in inflammatory response, such as leukocyte and lymphocyte activation in immune response, lymphocyte proliferation, mononuclear cell and T cell proliferation, leukocyte chemotaxis, etc., consistent with findings from previous studies highlighting immune dysfunction in PCOS [26]. In addition, the molecular function (MF) related genes were mainly enriched in immune system and inflammation-related functions like cytokine binding, cytokine receptor activity, toll-like receptor binding, G protein-coupled chemoattractant receptor, and chemokine receptor activities. The association between PCOS and inflammation was further emphasized as the GO analysis showed that DEGs related to specific cellular components (CC) were associated with immune system responses like immunological synapse, canonical inflammasome complex, alpha–beta T cell receptor complex, immunoglobulin complex, circulating, and secretory granule membrane and lumen. Studies have shown that women with PCOS have higher levels of inflammatory markers, such as excessive TNF-alpha from macrophages, while having lower anti-inflammatory adiponectin [2]. Further, hyperandrogenism in PCOS also elevates inflammatory markers such as interleukin (IL)−22, IL-1, and IL-6 [2]. These pro-inflammatory markers interfere with insulin signaling, causing insulin resistance and further worsening the PCOS conditions by increasing androgen production by ovarian theca cells [27]. In PCOS, chronic low-grade inflammation is not just a consequence but a driver of core dysfunctions, especially insulin resistance and hyperandrogenism. Therefore, these findings underscore the involvement and contribution of immune dysfunction and chronic inflammation to the pathophysiology of PCOS.

### Gene set enrichment analysis of differentially expressed gene (DEG)

The GSEA analysis revealed an increase in the KEGG pathways such as endocytosis, autophagy, ubiquitin-mediated proteolysis, and pathways majorly related to immune dysfunction, inflammation, and oxidative stress. According to the previous findings, autophagy appears to be increased in PCOS ovarian tissues of both human and rat model [28]. Similarly, multiple pathways associated with neurodegenerative diseases such as Alzheimer’s and Parkinson’s [29], and cardiovascular disease-diabetic cardiomyopathy [2] have also been associated with PCOS.

Further, network analysis of the association of KEGG pathways and genes related to the pathway such as regulation of mast cell activation plays a role in stress-induced inflammatory responses and is also related to sex hormones [30]. In addition, genes associated with multiple neurodegenerative diseases were also overexpressed in PCOS, as SNARE proteins play a crucial role in hormone secretion and neurotransmitter release, with SNAP-25 being a key gene involved in these processes [31]. The overexpression of components of SNARE protein complexes is related to obesity and diabetes, as they can impair the GLUT4 trafficking, which facilitates glucose to muscles and adipose tissue. Thus, overexpression of SNARE protein complexes can induce insulin resistance and hyperlipidemia in the body, which is closely related to the occurrence of PCOS [32]. Especially, overexpression or abnormal expression of SNAP-25 has been linked to insulin resistance, as SNAP-25 acts as a physiological brake to impair insulin action, while attenuation or deficiency of SNAP-25 results in higher insulin sensitivity [33, 34]. Similarly, higher expression of inflammation and oxidative stress also induce low-grade systemic inflammation, which is characteristic of obesity and hyperlipidemia [35]. Such exacerbated conditions further contribute to the pathogenesis of PCOS, given that insulin resistance and obesity are key etiological factors associated with the disorder [2].

While these findings provide valuable insights, the study is limited by the relatively small sample size and lack of experimental validation, such as qRT-PCR, which should be addressed in future investigations.

## Conclusion

In the present study, we analyzed the transcriptomic data of the ovarian granulosa cells of the PCOS-affected women. The study highlights that the gene expression in PCOS was found to have higher expression of inflammatory genes (SPI1, HSPB1, MNDA, and ITGAX) and androgen-mediated genes (SPI1 and ETS transcription factor), which showed the PCOS pathology. Analysis also indicates an enrichment of inflammation-associated pathways, including cytokine signaling, toll-like receptor pathways, and chemokine activity. Furthermore, immune system dysregulation was evident with increased lymphocyte and leukocyte activation and proliferation, reinforcing the contribution of immune dysfunction in PCOS. PCOS group also had a higher expression of TNFRSF1B genes related to hyperlipidemia and insulin resistance, which further fuel the PCOS by inducing obesity. The identified genes and enriched pathways could serve as potential drug targets. Inhibitors or modulators of these inflammatory or metabolic pathways, such as those of SNAP-25, may be developed to mitigate PCOS-associated inflammation, improving ovarian function and metabolic outcomes. In addition, the differentially expressed genes, especially those strongly associated with immune cell activation, may serve as non-invasive biomarkers for early diagnosis, risk stratification, or treatment monitoring in PCOS patients.

## Supplementary Information

Supplementary Material 1: Supplementary information. Supplementary Table S1. Gene IDs. Supplementary Table S2. Feature count after filtering. Supplementary Table S3. Initial count after filtering. Supplementary Table S4. DEGs detected. Supplementary Table S5. All GO.

Supplementary Material 2: Supplementary Table S6. Samples meeting the above inclusion criteria.

## Data Availability

The Next-Generation Sequencing (NGS) data files used to perform this study are available at the National Center for Biotechnology Information (NCBI) with BioProject ID PRJNA762274 and PRJNA576231.
